# The HIV-1 Antisense Protein (ASP) induces CD8 T cell responses during chronic infection

**DOI:** 10.1186/s12977-015-0135-y

**Published:** 2015-02-10

**Authors:** Anne Bet, Emmanuel Atangana Maze, Anju Bansal, Sarah Sterrett, Antoine Gross, Stéphanie Graff-Dubois, Assia Samri, Amélie Guihot, Christine Katlama, Ioannis Theodorou, Jean-Michel Mesnard, Arnaud Moris, Paul A Goepfert, Sylvain Cardinaud

**Affiliations:** Department of Microbiology, University of Alabama at Birmingham, 35294 Birmingham, AL USA; Department of Medicine, University of Alabama at Birmingham, 35294 Birmingham, AL USA; Sorbonne Universités, UPMC Université Paris 06, Center for Immunology and Microbial Infections - CIMI-Paris, F-75013 Paris, France; INSERM, U1135, Center for Immunology and Microbial Infections - CIMI-Paris, F-75013 Paris, France; CNRS, ERL 8255, Center for Immunology and Microbial Infections - CIMI-Paris, F-75013 Paris, France; CPBS, CNRS, UM5236, Université Montpellier, Montpellier, France; AP-HP, Hôpital Pitié-Salpêtière, Department of Immunology, F-75013 Paris, France; Sorbonne Universités, UPMC Univ Paris 06-UMR_S 1136 Pierre Louis Institute of Epidemiology and Public Health, F-75005 Paris, France; INSERM-UMR_S 1136 Pierre Louis Institute of Epidemiology and Public Health, F-75013 Paris, France; AP-HP, Groupe hospitalier Pitié Salpêtrière, Service de maladies infectieuses, Paris, F-75013 France; Present address: Hôpital Henri Mondor, Université Paris-Est Créteil, IMRB, Inserm U955 Equipe 16, labex Vaccine Research Institute (VRI), 51, avenue du Maréchal de Lattre de Tassigny, F-94010 Créteil Cedex, France

**Keywords:** Chronic HIV-1, ASP, CD8+ T-lymphocytes, HLA-I, Cryptic epitopes, Alternative reading frame

## Abstract

**Background:**

CD8+ T cells recognize HIV-1 epitopes translated from a gene’s primary reading frame (F1) and any one of its five alternative reading frames (ARFs) in the forward (F2, F3) or reverse (R1-3) directions. The 3’ end of HIV-1’s proviral coding strand contains a conserved sequence that is directly overlapping but antiparallel to the *env* gene (ARF R2) and encodes for a putative antisense HIV-1 protein called ASP. ASP expression has been demonstrated *in vitro* using HIV-transfected cell lines or infected cells. Although antibodies to ASP were previously detected in patient sera, T cell recognition of ASP-derived epitopes has not been evaluated. We therefore investigated the *ex vivo* and *in vitro* induction of ASP-specific T cell responses as a measure of immune recognition and protein expression during HIV-1 infection.

**Results:**

A panel of overlapping peptides was initially designed from the full-length ASP sequence to perform a global assessment of T cell responses. Recognition of ASP-derived antigens was evaluated in an IFN-γELISpot assay using PBMCs from HIV-1 seropositive and seronegative individuals. Eight of 25 patients had positive responses to ASP antigens and none of the seronegative donors responded. As a complimentary approach, a second set of antigens was designed using HLA-I binding motifs and affinities. Two ASP-derived peptides with high predicted binding affinities for HLA-A*02 (ASP-YL9) and HLA-B*07 (ASP-TL10) were tested using PBMCs from HIV-1 seropositive and seronegative individuals who expressed the matching HLA-I-restricting allele. We found that HLA-I-restricted ASP peptides were only recognized by CD8+ T cells from patients with the relevant HLA-I and did not induce responses in any of the seronegative donors or patients who do not express the restrictive HLA alleles. Further, ASP-YL9-specific CD8+ T cells had functional profiles that were similar to a previously described HLA-A*02-restricted epitope (Gag-SL9). Specific recognition of ASP-YL9 by CD8+ T cells was also demonstrated by tetramer staining using cells from an HLA-A*02 HIV-infected patient.

**Conclusion:**

Our results provide the first description of CD8+ T cell-mediated immune responses to ASP in HIV-1-infected patients, demonstrating that ASP is expressed during infection. Our identification of epitopes within ASP has implications for designing HIV vaccines.

**Electronic supplementary material:**

The online version of this article (doi:10.1186/s12977-015-0135-y) contains supplementary material, which is available to authorized users.

## Background

Thirty years after its appearance, the HIV/AIDS epidemic still represents a major public health threat worldwide. Strategies for developing effective host immunity and protection against HIV-1 infection or lifelong control of the virus are urgently needed. Multiples lines of evidence suggest that HIV-specific cytotoxic CD8+ T cells (CTLs) participate in controlling viral replication [1-3]. During acute infection, the expansion of HIV-specific CTLs occurs prior to the appearance of neutralizing antibodies and is associated with decreased viremia [4,5]. Delayed disease progression has been correlated with the detection of Gag-specific CTLs and the presence of protective human leukocyte antigen class I (HLA-I) alleles, which effectively present HIV-1 derived epitopes for immune recognition [6-10]. However, HIV rapidly mutates to evade virus-specific T cell responses, underlying the selection pressure exerted by CTLs [11-13]. Thus, protection against HIV will likely require a vaccine that affords immediate CTL recognition of infected cells through sustained presentation of conserved T cell epitopes.

CTLs are stimulated by peptides presented by MHC-I molecules and generated from the proteolysis of all nine proteins encoded by the three forward reading frames of its sense mRNA strand. In addition to protein-derived epitopes, investigations over the last two decades have led to the discovery of cryptic epitopes, a novel set of T cell targets translated from alternative reading frames (ARFs) in both sense and antisense transcripts during autoimmune disease, cancer and several viral infections, including HIV-1 and SIV [14-26]. Moreover, sequence analysis of HIV-1 clade B isolates have repeatedly detected a conserved open reading frame in an ARF of *env*, suggesting the existence of a possible tenth viral protein [27-32]. Vaquero *et al*. demonstrated *in vitro* that this antisense ARF encodes a protein of 19kD, so called antisense protein (ASP) [33]. Subsequently, the existence of ASP has been supported by its *in vitro* expression from a native upstream promoter and, shortly thereafter, the detection of antibodies to ASP in patient sera [28,33,34]. Recently, we and others have confirmed that ASP is expressed by several cell types during HIV-1 infection [29,35-38]. Interestingly, ASP was detected in monocyte-derived macrophages and dendritic cells, which was consistent with preferential transcription of the antisense strand in these antigen-presenting cells (APC) [37]. Collectively, the aforementioned studies suggest that ASP is produced during the viral cycle. We previously demonstrated that ARF-derived HIV-1 antigens (i.e. cryptic epitopes, CE) are produced during infection by detecting CE-specific CTLs [17]. We therefore extended this work by evaluating T cell recognition of ASP-derived antigens in HIV-1 seropositive patients.

In an unbiased approach, we used pools of overlapping ARF peptides spanning the entire length of ASP to detect ASP-specific T cell responses in PBMCs obtained from HIV-1 seropositive and seronegative donors. We further investigated CD8+ T cell responses to predicted HLA-I-restricted epitopes derived from ASP. Overall, our findings show that ASP is specifically targeted by CD8+ T cells of chronically infected patients, revealing ASP as a HIV-1 antigen.

## Results

### ASP-overlapping peptides induce IFN-γ T cell responses in HIV seropositive patients

**Figure 1:**
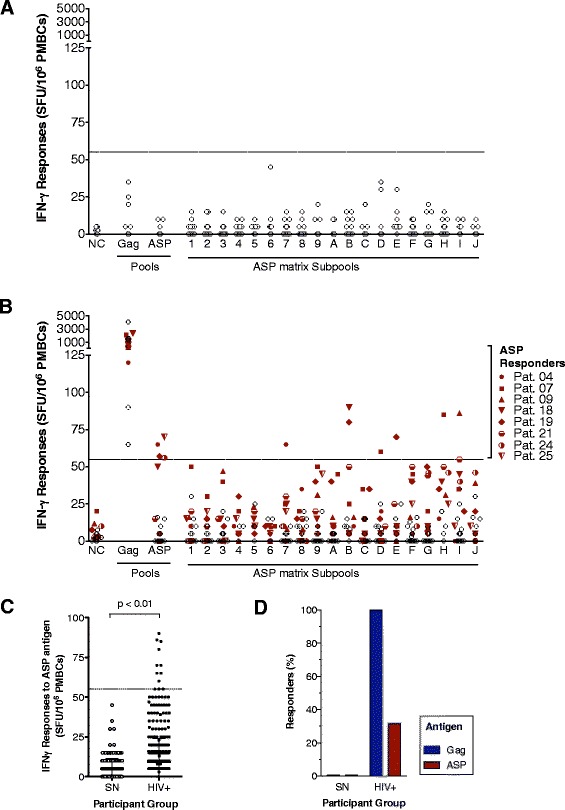
**HIV-1 ASP peptide pools activate IFN-γ T cell responses in a cohort of HIV+ individuals.** The magnitude of IFN-γ ELISpot responses (SFU/10^6^ PBMCs) to the Gag or ASP pools and subpools (1 to 9, and A to J) are shown for **(A)** seronegative donors and **(B)** HIV-1+ patients. Each dot represents one donor and mean IFN-γ responses of triplicate or quadruplicate are indicated. The dashed line marks the standardized threshold (55 SFU/10^6^ PBMCs) for positivity. **(C)** PBMCs were assessed for IFN-γ production using ASP pools and 19 ASP subpools. Mann–Whitney U tests were performed to determine significant differences between the median responses to peptide pools (p < 0.01). **(D)** The frequency of responders is illustrated as a proportion of seronegative (SN) or seropositive (HIV+) individuals with positive responses to the Gag pool (blue column) and either to the ASP pool or any ASP subpool (red column).

Immune responses to ASP were first studied using PBMCs from HIV-1 subtype B seropositive patients (Pats.1 to 25, Additional file 1: Table S1) who were off antiretroviral therapy (ART) and 10 seronegative donors (SN). T cell responses were measured with an *ex vivo* interferon gamma (IFN-γ)-ELISpot assay using a pool of peptides encompassing the whole ASP sequence (85 peptides, 14-18-mers overlapping by 10). To increase the likelihood of detecting CTL responses primed during acute or chronic infection, the ASP pool included peptides derived from the sequence of a transmitter founder virus WITO_TF1 [39] and NL4-3, a viral strain isolated during chronic infection and expressing ASP in infected cells [29,33,37,38]. Within this cohort, 4 out of the 25 patients responded to ASP (Figure 1B, Pats.04, 19, 24, 25). A pool of peptides encompassing Gag was used as a positive control and induced CTL responses in all patients. No ASP-specific or Gag-specific responses were detected in any seronegative donors (Figure 1A). We then analyzed T cell responses to 19 subpools of ASP, containing 8 to 10 peptides (Additional file 2: Figure S1). We observed that 6 patients (Figure 1B, Pats 04, 07, 09, 18, 19, 21) responded to one or two subpools of ASP peptides and that a total of 6 different subpools induced CTL responses (#7, B, D, E, H, I). Thereafter, two patients (Pats.04 and 19) responded to both the total pool of ASP peptides as well as one or more subpools (subpools #7 and B, E). Notably, Pats.24 and 25 responses to subpools (G, H, J, and 9, respectively) tended to be positive though just under our stringent limit of positivity (more than four times background but 45 SFU/10^6^ PBMCs). Overall, the magnitude of T cell responses to the ASP pool or subpools was significantly greater in seropositive patients than in seronegative donors (p < 0.01; Figure 1C). Overall, we found a high frequency of patients responding to ASP antigens (32%; Figure 1D).

### Identification of ASP-specific HLA-A*02 and –B*07-restricted T cell responses

**Figure 2:**
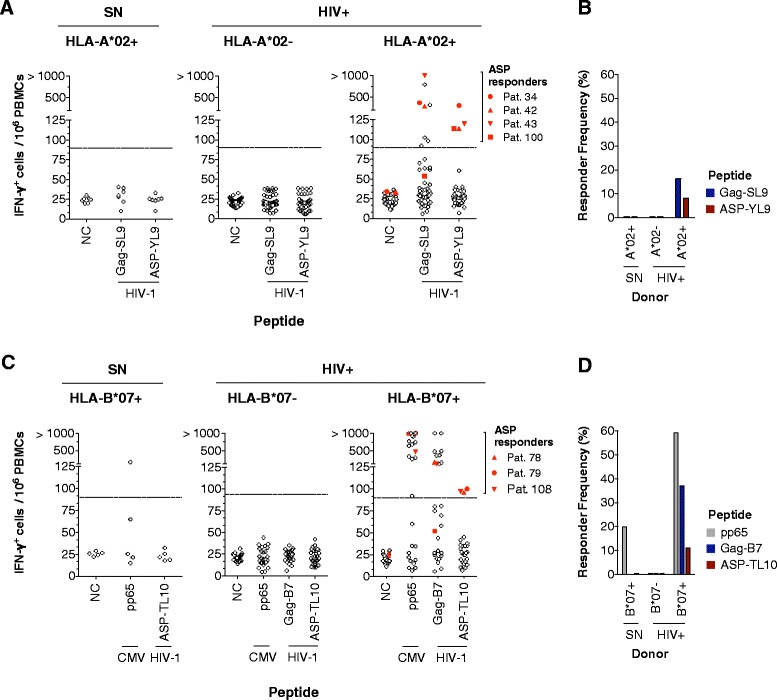
**HLA-I-restricted ASP epitopes are recognized by CD8+ T cells during HIV-1 infection. (A)** PBMCs isolated from HIV-1 seropositive patients and seronegative donors were stimulated *ex vivo* with ASP-YL9 in an IFN-γ ELISpot assay. An HCV irrelevant and HIV Gag-derived HLA-A*02-restricted (Gag-SL9) peptide was used as negative and positive controls, respectively. Dotted line indicates a mean threshold of peptide responders (4 times background, 90 SFU/10^6^ PBMCs). Four HLA-A*02+ HIV+ patients had responses to ASP-YL9 (n = 49, right panel), whereas no IFN-γ secretion was detected in HLA-A*02- HIV+ patients (n = 30, middle panel) or HLA-A*02+ seronegative donors (SN, n = 7, left panel). **(B)** Frequencies of ASP-YL9-, or Gag-SL9- responders according to each group are shown. **(C)** As in (B) with the ASP-TL10 peptide and a CMV-derived (pp65) peptide. Three HLA-B*07+ HIV+ donors were responding to ASP-TL10 (n = 27, right panel), whereas no responses were detected in HLA-B*07- seropositive donors (n = 27, middle panel) and HLA-B*07+ SN (n = 5, left panel). **(D)** Frequencies of donors responding to ASP-TL10, HIV Gag (Gag-B7) or CMV (pp65) are indicated according to each group.

Our panels of OLP showed that HIV-1-specific IFN-γ T cell responses were induced by ASP-derived antigens. Attempts to further map ASP subpool responses failed to consistently identify individual epitopes or peptides that could contain them. However, the analysis was performed using 14-18-mer peptides for global sequence coverage of the ASP protein and may have provided suboptimal targets for CD8+ T cells, which recognize MHC-I molecules presenting 8-11-mer peptides. We therefore investigated ASP-specific T cell responses using a common “reverse immunology” approach [17]. In brief, potential ASP-derived peptides with high affinity to a specific HLA-I allele were predicted, and T cell recognition of these candidates was evaluated in HLA-I-matched individuals. The analysis of HIV-1 clade B and M sequences revealed two peptide sequences, ASP-YL9 (YLYNSLLQL) and ASP-TL10 (TPNGSIFTTL), with high binding scores to HLA-A*02 and HLA-B*07 molecules, respectively (Additional file 3: Table S2). In comparing the conservation of classical epitopes and ASP peptides, both ASP peptides had classical equivalents that were either poorly (*e.g*. ASP-TL-10 and Gag-YF10) or highly (*e.g*. ASP-YL9 and Env-KL9) conserved [40-42]. To increase the probability of detecting HLA-I-restricted CTL responses to ASP, the ASP-YL9 and ASP-TL10 peptides were selected for use in immunoassays based on their high HLA-binding score and relative conservation. T cell responses against these peptides were investigated within a cohort of HIV-1 seropositive donors (Pats.32 to 126, Additional file 1: Table S1). PBMCs were screened for HLA-A*02 and HLA-B*07 alleles expression by antibody staining. PBMCs from 49 HLA-*02+ patients (Pats.32 to 74, 98 to 103) were assayed for *ex vivo* IFN-γ ELISpot responses against the ASP-YL9 peptide. A known HIV-derived HLA-A*02-restricted epitope (Gag-SL9) and an HCV-derived irrelevant peptide were used as positive and negative controls (NC), respectively (Figure 2A). Four patients (Pats.34, 42, and 43 and 100) responded to ASP-YL9. Magnitudes of ASP-specific T cell responses ranged from 114 to 307 SFU/10^6^ PBMCs and were weaker than Gag-SL9 responses (92 to more than 1000 SFU/10^6^ PBMCs). Strikingly, no responses against ASP-YL9 were detected in HLA-A*02+ HIV-1 seronegative donors and in HLA-A*02 negative patients (n = 30, Pats.89 to 95, 104 to 126). Height percent of PBMCs from HLA-A*02+ seropositive donors were activated by ASP-YL9 (Figure 2B). We then assessed *ex vivo* IFN-γ responses against the ASP-TL10 peptide. We used a pool of three known HLA-B*07-restricted Gag epitopes (pool named as “Gag-B7”) and a CMV-derived HLA-B*07-restricted epitope (pp65) as positive controls. Twenty-seven HLA-B*07+ patients were tested (Pats.69 to 88, 104 to 110) and three individuals (Pats.78, 79 and 108) positively responded to the ASP-TL10 antigen (average magnitude of responses of 98 SFU/10^6^ PBMCS) (Figure 2C). No ASP-TL10-specific T cell responses were found within the HLA-B*07+ seronegative donors and HLA-B*07 negative HIV patients (n = 27, Pats.66 to 68; 96 to 103, 111 to 126). Eleven percent of PBMCs from HLA-B*07+ seropositive donors were activated by ASP-TL10 (Figure 2D). Taken together, the frequency of responders to ASP-YL9 or ASP-TL10 was lower than the proportion of patients who responded to immunodominant Gag-derived antigens: 16% of HLA-A*02+ patients responded to Gag-SL9 and 37% of HLA-B*07+ patients responded to Gag-B7.

### ASP-specific CD8+ T cells produce multiple cytokines and chemokines

**Figure 3:**
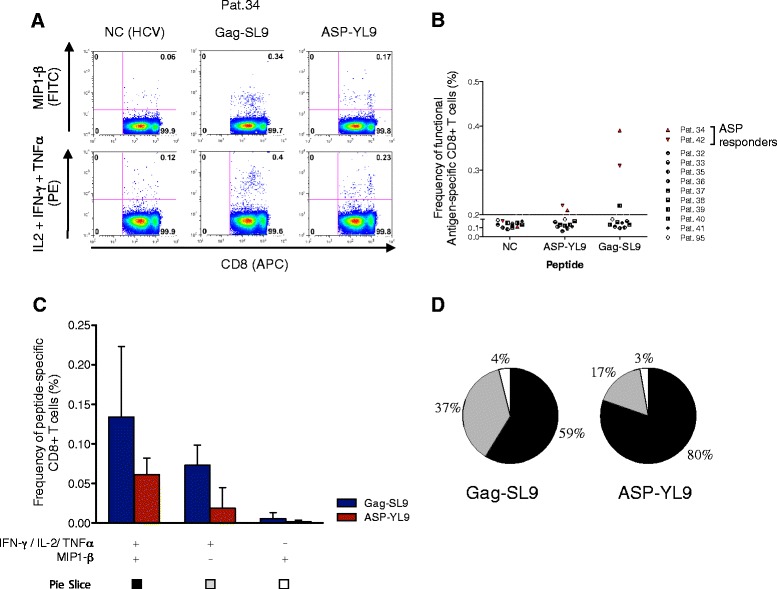
**The HIV-1 ASP-YL9 peptide activates CD8** ^**+**^ **T cell responses.** Intracellular cytokine staining (ICS) was used to analyze CD8+ T cell responses to ASP-YL9 in 11 HLA-A*02+ patients (Pats.32 to 42) and one HLA-A*02- patient (Pat.95). **(A)** Flow cytometry analysis from Pat.34, illustrating MIP1-β versus IL-2/TNFα/IFN-γ production by CD8+ cells after HCV- (negative control, NC), HIV ASP-YL9 or HIV Gag-SL9 stimulation. **(B)** Frequencies of CD8+ cells producing at least one of the analyzed cytokine/chemokine. Pats. 34 and 42 were activated upon ASP-YL9 stimulation. **(C)** For these two patients, percentages of Gag-SL9 (blue) or ASP-YL9 (red) stimulated CD8+ cells, producing either only MIP1-β (white), only IL-2/TNFα/IFN-γ (grey), or both chemokines/cytokines panels (black) were analyzed. NC background was subtracted and average percentages (±SD) from Pats.34 and 42 analysis are presented. **(D)** Pies indicate relative proportions of Gag-SL9 or ASP-YL9 activated CD8+ T cells producing each panel of cytokines/chemokines (presented in C).

Recognition of 8-11-mer peptides by T cell receptors of CD8+ T cells requires presentation of these antigens by HLA-I molecules. To assess whether CD8+ T cells recognize the ASP-Y9L peptide, we analyzed production of intracellular cytokines in a flow cytometry assay (ICS) following stimulation of PBMCs from 11 HLA-A*02+ patients (Pats.32 to 42), and one HLA-A*02- patient as control (Pat.95). Cells were pulsed with the ASP-YL9 peptide. Production of MIP1-β or IL-2, TNFα and IFN-γ together was assessed on CD8+ cells (Figure 3A). We measured the proportion of activated cells producing at least one of these cytokines/chemokines upon peptide stimulation (Figure 3B). 0.1% of CD8+ T were specifically stimulated by ASP-YL9 for two HLA-A*02+ patients (Pats.34 and 42). No ASP-specific stimulation of CD8+ T cells was observed for Pat.95 (HLA-A*02-). Induction of Gag-SL9-specific CD8+ T cells was also detected in three patients, including Pats.34 and 42 (range, 0.1 to 0.3% of CD8+ T cells). The results strongly suggest that the IFN-γ detected in our previous ELISpot assays was secreted by CD8+ T cells (Figure 2A, B). We then evaluated whether ASP-specific CD8+ T cells were producing multiple cytokines together (Figure 3C). 97% of ASP-YL9-specific CD8+ T cells were producing IL-2, IFN-γ or TNFα, together with a production of MIP1-β for 80% of them (Figure 3D, black pie). Thus, epitope-specific CD8+ T cells produce multiple cytokine/chemokines upon recognition of ASP-YL9. This subset exhibits a functional profile similar to Gag-SL9-specific CD8+ T cells, with a majority of cells producing multiple cytokine/chemokines at the same time (Figure 3D). Although the measurable cytokines are not evidence of T cell-mediated cytotoxicity, the capacity of ASP-specific CD8+ T cells to produce multiple cytokines/chemokines possibly enhances their ability to kill HIV-1-infected cells [43,44].

### Identification of ASP-YL9-specific CD8+ T cells by tetramer staining

**Figure 4:**
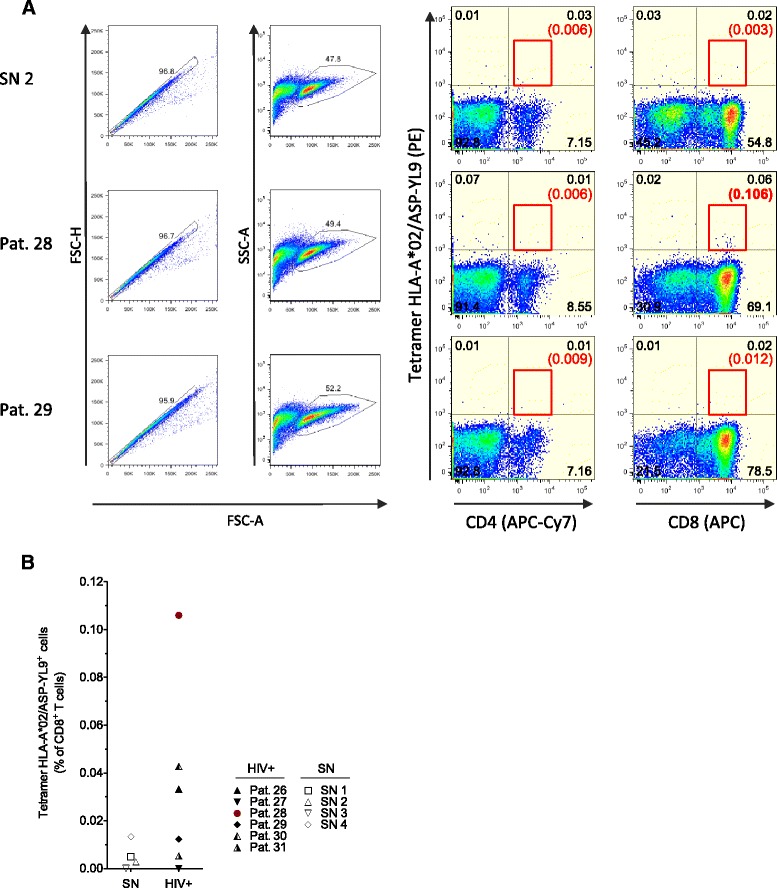
Detection by tetramer of HLA-A*02-restricted, ASP-YL9-specific CD8+ T cells in HIV+ patients. Following *in vitro* restimulation of PBMCs with ASP-YL9 peptide, cell lines from 6 HLA-A*02+ HIV+ patients (Pats. 26 to 31) and 4 HLA-A*02+ seronegative donors (SN 1 to 4) were stained for CD4 and CD8 in combination with the HLA-A*02/ASP-YL9 tetramer. **(A)** Gating strategies and illustration of tetramer staining for SN2, Pat.28 and Pat.29. In Pat.28, 0.06% tetramers + cells were detected corresponding to 0.11% of CD8+ T cells. No tetramer + cells were detected among CD4+ cells. In Pat.29, no significant tetramer+ population was detected (Fisher-exact test). **(B)** Percentages of HLA-A*02/ASP-YL9 tetramer+ cells within CD8+ T cells of HIV+ patients and seronegative donors.

We further examined the antigen specificity of CD8+ T cell responses in a subset of participants (Pats.26 to 31). We previously showed that magnitude of HIV-1 ARF-specific CD8+ T cell responses are increased following *in vitro* stimulation of PBMCs with antigens [45]. PBMCs from HLA-A*02+ HIV+ patients and seronegative control donors (SN) were expanded with the ASP-YL9 peptide and stained with an ASP-YL9/HLA-A*02 tetramer. When PBMCs from Pat.28 were analyzed, 0.11% of CD8+ T cells were stained specifically with the HLA-A*02/ASP-YL9 tetramer, corresponding to 0.06% of the total T cell population (Figure 4A, B). In contrast, no specific staining could be detected within the CD4+ T cell population (Figure 4A). No tetramer staining was detected in other patients and seronegative donors. Together, these results indicate that the ASP-YL9 peptide elicits HLA-A*02 restricted CD8+ T cells during HIV infection.

## Discussion

The existence of a tenth HIV-1 protein encoded by genomic plus strand has been suggested since the late 80s [27]. Yet, *in vivo* antisense transcription and translation of not only HIV-1, but also of other retroviruses, has remained a topic of debate. In this study, we show that CD8 T cell responses to an antisense derived HIV protein (ASP) are detected during chronic HIV infection. Further, we demonstrate that these ASP specific CD8 T cells have comparable functionality to the traditional protein-derived HLA-I restricted epitopes.

Virological but also immunological attributes of antisense proteins were emphasized by HBZ and APH-2, two antisense strand-encoded proteins from the human T cell leukemia virus type 1 (HTLV-1) and HTLV-2, respectively [46-48]. HBZ modulates viral transcription, persistence of the virus and infectivity. It may also play a role in induction of cellular immune responses controlling the virus [49]. Thus, antisense proteins appear critical for understanding retroviral pathogenesis. The role of antisense transcripts from HIV-1 still remains poorly studied. Their existence was demonstrated *in vitro* and in the context of infection [32,50]. Conservation of the antisense reading frame directed investigations to search for a protein translated by these antisense transcripts [27,35,38]. Vaquero *et al.* demonstrated that antibodies from sera of seropositive individuals were detecting ASP [33]. Evidence of the expression of ASP was further confirmed by *in vitro* experiments [29,34,37]. Although all these approaches stressed the fact that ASP is expressed during infection, studies have not convinced the scientific community of the existence of this protein. In the present study, we revisited this question by investigating the induction of ASP-specific CD8+ T cells in patients after HIV-1 infection. Our results show for the first time that a proportion of HIV-specific CD8+ T cells are targeting ASP and are activated during chronic infection.

Over the last three decades, the pool of HIV-1 CTL epitopes has been studied extensively. CTLs target epitopes from all nine structural and regulatory proteins encoded by open reading frames in the RNA sense strand. Alternative translation of retroviral genes was initially hypothesized in mice infected by LMP5, an immunodeficiency-causing retrovirus [51]. Green and colleagues expanded the known pool of T cell targets by demonstrating that CTL epitopes were generated *in vivo* from translation of a *gag* ARF, rather than the gene’s primary reading frame. ARF-derived antigens, often smaller in size than traditional proteins, appear labile and difficult to detect by conventional approaches. Nevertheless, the incredible sensitivity and specificity of CTLs allows for efficient detection of cryptic epitopes (CE) from ARFs. CTLs targeting CE of HIV-1 and SIV have been detected by several groups, thus revealing the translation of sense and antisense ARFs [15-17,52,53]. CTLs targeting CE are elicited during infection following stimulation by antigen-presenting cells. Further, mutations within these CE can arise in order to escape CTL responses [11,53]. All of these findings strongly suggest that ARF sequences are translated during the viral cycle and subsequently recognized by the immune system. Here, we demonstrated that ASP-specific CD8+ T cells were detectable in the blood of seropositive individuals by combining two complementary approaches. Strikingly, no epitope-specific T cell responses were detected in seronegative donors or in HIV+ patients who did not carry the appropriate HLA-I alleles. Peptides from various locations within ASP were recognized. Our results indicate that the full-length ASP protein is likely processed and presented by MHC-I molecules. Since ASP-specific CD8+ T cells are generated in infected individuals during the course of HIV-1 infection, our results strongly suggest that ASP is expressed by infected cells and constitutes a source of antigens for inducing CD8+ T cell responses, either following natural infection or vaccination.

In our unbiased approach, approximately 30% of the donors showed positive IFN-γ responses to ASP, whereas the response level to individual peptides ASP-YL9 and ASP-TL10 were detected in 8% of HLA-A*02+ donors and 11% of HLA-B*07+ donors, respectively. The proportion of responses to pool and subpools may have been higher than the proportion of responses to optimal peptides because pools include a greater number of peptides, thus increasing the likelihood of activating ASP-specific CTLs with various antigenic specificities. Our lead hypothesis was that ASP is expressed during infection and induces CD8+ T cell responses. However, whether ASP is expressed during acute and/or chronic infections remains to be determined. Thereafter, we thought that including peptides overlapping WITO_TF1 sequence might increase the chance to activate CTL responses primed during the early step of acute infection. In addition, NL4-3, that was used to design the set of peptides overlapping a viral strain isolated during chronic infection, was selected because we have previously shown that NL4-3-infected cells express ASP [29,37,38]. WITO_TF1 and NL4-3 might not be representative of the circulating virus present in the tested patients but allowed us to maximize our chance to detect CTL responses. Obviously, it would be of great interest to sequence the quasi-species circulating in the blood of patients to study the conservation of ASP ORF and amino acid sequences with regards to ASP-specific CTL responses.

As majority of our patients were treated, the frequency of ASP responders was doubtlessly underestimated. ART may impact the magnitude and the breadth of ASP-specific CTL responses, as demonstrated for ARF sense and antisense epitopes [54]. The magnitude of T cell responses to ASP appear weak but were significantly higher that non-specific responses from seronegative donors. Although responses to the ASP peptide pool and subpools did not exceed to 100 SFU/10^6^ PBMCs, ASP-YL9 responses reached 300 SFU/10^6^ of PBMCs. The capacity of peptide pools to activate T cell responses was evaluated using cryopreserved PBMCs, whereas fresh PBMCs were used to detect ASP-YL9 and ASP-TL10 responses. Although the magnitudes of CTL responses might have been stronger using fresh samples, using frozen cells, we readily detected ASP-specific CTL responses. Magnitudes of ASP-specific responses from *ex vivo* PBMCs are in fact consistent with previous analyses for responses to cryptic epitopes from ARFs [15-17,45]. However, the limited magnitude of T cell responses may be due to low expression of these antigens, poor MHC-I binding and presentation, or a T cell repertoire with a low affinity for these antigens. Indeed, in contrast to Gag, cryptic epitopes are produced by non-conventional mechanisms of translation of the viral genome. To our knowledge, events responsible for protein synthesis from ARF of transcripts and mechanisms regulating this process are not clearly defined. The transcription factors NFκB and Sp1 but not the HIV-1 Tat protein are positive modulators of HIV antisense transcription [55]. Antisense RNA appeared preferentially transcribed in specific cell-types (*e.g.* monocyte-derived cells) [37]. ASP potentially induces autophagy [38]. Thus, various parameters may justify presumed low abundance of ASP in infected cells, and therefore a weak induction of CTL responses. HIV-specific CTLs conferring protection to patients have been shown to have a high sensitivity to antigen, develop multiple antiviral functions and have non-exhausted phenotype [56]. In our approach, we showed by intracellular staining that production of cytokine/chemokines by ASP-specific CTLs appears as diverse as for conventional CTLs (Gag-SL9). The quality of ASP-specific CTLs responses, their phenotypes and capacity to control infection remains to be defined.

We focused our explorations on individuals expressing HLA-A*02 or HLA-B*07, two alleles frequent among the Caucasian population and whose peptide anchor motifs are well defined. These alleles are not associated with a delayed disease progression. It would be of interest to analyze ASP-specific CTL responses in patients that spontaneously control the infection but also in post-treatment controllers [57]. Current vaccines do not prioritize the expression of ASP or epitopes from this ARF. Harnessing the strategic engineering and control of HIV-1’s genetic code in the context of a highly efficient vector has the potential to increase production and presentation of viral immunogens following vaccination. Importantly, the number of vector-derived immunogens could be increased through constructs that express both sense and antisense reading frames, thus inducing broader immune responses to protein- and ARF-derived epitopes without expanding the size of the vector insert [5,58-63]. In fact, vector-derived cryptic epitopes encoded by antisense *gag* and *pol* ARFs have been shown to induce HIV-1-specific T cell responses in vaccinees who participated in a Phase 2b trial (HVTN 205). This suggests that current MVA/DNA vaccines encoding the *env* region may already induce ASP-specific CD8+ T cell responses [45].

## Conclusions

Our data demonstrated for the first time that epitope-specific CD8+ T cells from infected individuals target ASP. Moreover, the *ex vivo* induction of ASP-specific CD8+ T cell responses provides strong evidence of ASP expression during infection, thereby supporting ARFs as an additional source of HIV-1 immunogens. Future studies assessing the functionality of ASP and other conserved ARF sequences will therefore be critical for enhancing our understanding of viral life cycle and developing effective HIV-1 vaccines.

## Methods

### Study populations

Two cohorts of HIV infected patients were studied. First, peripheral blood mononuclear cells (PBMCs) were collected from US patients with HIV-1 clade B infections (Additional file 1: Table S1, Pat 1 to 25) and seronegative donors (*n* = 10). All HIV seropositive patients were off ART and, with a median CD4 T cell count of 789 cells/μL (range, 449 – 1176 cells/μL), median CD4 nadir of 434 cells/μL (range, 116 – 827 cells/μL), and median plasma viral load (VL) of 616 RNA copies/mL (range, 21 – 16,400 RNA copies/mL). HLA class I alleles were typed to two- or four-digit specificity by PCR amplification using sequence-specific primers as described before [64].

Second, PBMCs were collected from HIV-1 positive HCV (Hepatitis C virus) negative patients from a French ANRS cohort (Additional file 1: Table S1, Pats. 26 to 126) [65]. Ninety-two percent of these patients were on ART (range, 0.5 - 26 years). Twenty-five percent of all patients had a detectable viral load (range, 21–2,951,507 RNA copies/mL) and the median CD4 T cell count was 522 (range, 18 – 1854 cells/μL). All patients were chronically infected and 8 were recently detected as seropositive (Pats. 26, 42, 50, 70, 87, 100, 107, 126). HLA types were first determined by FACS analysis using anti-HLA*07 (BB7.1, AbD Serotec) and anti-HLA-A*02 (BB7.2, Biolegends) antibodies. HLA-B*07+ and HLA-A*02+ cell lines were simultaneously analyzed as positive controls. HLA status of ASP responders was further confirmed by genotyping using PCR or using the Luminex xMAP technology [66,67]. PBMCs from HIV-1–seronegative control donors were purchased from the Etablissement Français du Sang of the Pitié-Salpêtrière Hospital (Paris, France).

Demographic, HLA-typing, virological and clinical characteristics of patients included in both cohorts are shown in Additional file 1: Table S1.

### Ethics statement

All samples were obtained according to protocols approved by the Institutional Review Board (IRB) at the University of Alabama at Birmingham. Patient samples were collected according to French Ethical rules. Written informed consent and approval by institutional review Board at the Pitié-Salpêtrière Hospital were obtained.

### Antigen design

Consensus clade B 15-mer peptides (123 total) overlapping by 11 amino acids (aa) for HIV-1 Gag were obtained through the NIH AIDS Reagent Program (catalog #8117). In our unbiased approach, we used ASP sequences obtained from the transmitted founder virus of an acute patient (WITO_TF1, Genbank Acc.# JN944938.1) and a HIV-1 lab strain, NL4.3 (Genbank Acc.# M19921), to design 85 overlapping ASP peptides (OLPs; 14 to 18mers overlapping by 10 residues) using the Los Alamos National Lab’s PeptGen tool (http://www.hiv.lanl.gov/content/sequence/PEPTGEN/peptgen.html; Additional file 2: Figure S1). Peptides were synthesized in a 96 well array format by New England Peptide [68]. Individual OLPs were combined into a single Gag or ASP pool. ASP subpools of no more than 10 peptides were used in a matrix format. Peptides were reconstituted in 100% DMSO at 40 mM/peptide. Working stocks were prepared with water to a final concentration of 100 μM/peptide. The final concentration during the ELISpot assay was 5 μM/peptide in 100 μl, corresponding to ≤1% and ≤0.1% of DMSO (v/v) for the total pools (ASP and Gag) and ASP subpools, respectively.

As a complementary strategy, HLA-I binding affinities and associations of potential ASP epitopes were evaluated using BIMAS, SYFPEITHI and NetMHC prediction algorithms (Additional file 3: Table S2) [69-71]. Two epitopes were synthesized, YLYNSLLQL (ASP-YL9) and TPNGSIFTTL (ASP-TL10) (Proimmune). Peptides were selected based on high binding affinities for HLA-I alleles HLA-A*02 and HLA-B*07 respectively, and on the other hand on their conservation among HIV-1 strains. All peptides used in this study were > 85% purity, as shown by HPLC profiles.

### *Ex vivo* IFN-γ ELISpot assay

In the unbiased approach using OLP, cryopreserved PBMCs were thawed and rested overnight at 37°C, 5% CO_2_ in RPMI containing 5% human serum and supplemented with penicillin, streptomycin, and L-glutamine. Interferon gamma ELISpot assays were performed as previously described using a total pool containing all peptides, subpools, or individual peptides [15,45]. Antigens were tested in duplicate or triplicate with 100,000 PBMCs/well in nitrocellulose 96-well plates precoated overnight with anti-IFN-γ coating antibody and unstimulated cells were plated in quadruplicate as a negative control; phytohemagglutinin (PHA) was tested in duplicate as a positive control. Spot forming units (SFU) were counted by an ELISpot reader (ImmunoSpot; CTL) as a measure of cytokine production.

In the approach using optimal peptides, *ex vivo* ficoll-isolated PBMCs were stimulated by 1 μM synthetic peptides in IFN-γ ELISpot assays as previously described [72]. An HCV-derived epitope (GPRLGVRAT) was used as negative control (NC), and a CMV-derived epitope (pp65 _417_TPRVTGGGAM_426_) as positive control for HLA-B*07 donors. Known HIV-1- Gag-derived epitopes were used to test HIV reactivity, either a pool (Gag-B7) of three HLA-B*07-restricted epitopes (Gag-SV9, p24 _16_SPRTLNAWV_24_, Gag-TL9, p24 _48_TPQDLNTML_56_, Gag-YF9, p2p7p1p6 _121_YPLASLRSLF_130_) or an HLA-A*02-restricted epitope (Gag-SL9, p17 _77_SLYNTVATL_85_) [41,73].

T cell responses were reported as positive if i) the mean response to the sample’s negative control was inferior to 30 SFU/10^6^ PMBCs, ii) the average response was more than 55 SFU/10^6^ PMBCs, iii) greater than 4 times the mean response to the sample’s negative control, iv) greater than the mean response to the sample’s negative control plus 3 standard deviations, and v) the mean response to the sample’s positive control (PHA) was greater than 500 SFU/10^6^ PBMCs [74].

### Intracellular cytokine staining (ICS) assay

*Ex vivo* isolated PBMCs from 11 HLA-A*02+ and 1 HLA-A*02- HIV donors were rested overnight in RPMI 1640 supplemented with 5% human serum (Institut Jacques Boy), 2 mM L-glutamine, 100 IU/mL penicillin, 100 μg/mL streptomycin, 10 mM Hepes, 1% nonessential amino acids, 1 mM sodium pyruvate. Cells were pulsed with individual peptides (1 μM) for 2 hours at 37°C and brefeldin A (5 μg/mL; Molecular Probes) was added for 6 hours at 37 °C. Cells were fixed, permeabilized, and stained using standard procedures with CD8-APC (RPA-T8), TNFα-PE (MAb11), IL-2-PE (MQ1-17H12), IFN-γ-PE (B27) (BD Pharmingen), and MIP-1β-FITC (24006, R&D Systems). Cells were analyzed with a FACS-Calibur™ cytometer (BD Biosciences). Flow cytometry data were analyzed with FlowJo software (Tree Star).

### *In vitro* expansion of ASP-specific CD8+ T cells

Cryopreserved PBMCs from 6 HIV-1+ patients and 4 seronegative donors with HLA-A*02 alleles were pulsed with the ASP-YL9 peptide (1 μM) and cultured for 7 days in the presence of IL-2 (100 IU/mL; Miltenyi). On day 3, 20 ng/ml recombinant human IL-7 (Sanofi-Synthelabo) was added. Samples were then evaluated for antigen specificity in a flow cytometry-based assay using anti-CD4-APC-Cy7 (RPA-T4, BD Pharmingen), anti-CD8-APC (RPA-T8 BD Pharmingen), and PE-conjugated HLA-A*02 tetramers presenting the ASP-YL9 peptide (TC Metrix). Cultured cells were incubated in PBS 0.5% BSA (Sigma) with tetramers and antibodies for 20 min at 4°C, then washed and fixed before measuring the frequency of single CD4- tetramer + CD8+ T cells for each participant with a BD FACS Canto cytometer.

### Statistical analysis

T cell responses were analyzed for differences in magnitude using two-tailed non-parametric Mann–Whitney *U* tests calculated by GraphPad Prism v6.0b and frequency comparisons were done using two-tailed Fisher’s exact tests calculated by GraphPad QuickCalcs, where p values of 0.00001 or less were considered statistically significant. A high-powered statistical test and an adjusted significance threshold were applied to stringently assess small differences between large sample sizes (total number of subset and gated events) and Type I errors.

## Additional files

Additional file 1: Table S1. Demographic, HLA-typing, virological and clinical characteristics of patients. Additional file 2: Figure S1. Antigens designed for detection of epitope-specific T cell responses provide global sequence coverage of HIV-1 ASP. (A) Overlapping peptides were designed using ASP sequences isolated from transmitted founder virus of an acute patient (WITO_TF1, red) and a HIV-1 lab strain (NL4-3, green). Peptides encoded by both strains are indicated in purple. Amino acid differences between peptides are highlighted in bold. Translated sequences are shown in the 5’ to 3’ direction, corresponding to the N- and C-termini of ASP, respectively. Sequences for ASP-YL9 and ASP-TL10 peptides are boxed. ni: not included, if the peptide could not be synthesized or did not pass NEP’s quality control standards. (B) Matrix design for subpools ASP peptides (A to J, and 1 to 9). Peptide number refers to the peptides listed in (A). Patients responding to peptide subpools are indicated along the x- and right y-axis. Additional file 3: Table S2. Binding affinity and conservation of HLA-A*02- and HLA-B*07-restricted HIV-1 epitopes.

## Abbreviations

HIV-1: Human immunodeficiency virus type I
ASP: HIV-1 antisense protein
HLA-I: Human leukocyte antigen class I
MHC-I: Major histocompatibility class I molecule
ARF: Alternative reading frame
ORF: Open reading frame
OLP: Overlapping peptides
CE: Cryptic epitope
