# Supplementary material for: The HIV-1 Antisense Protein (ASP) induces CD8 T cell responses during chronic infection
Source: Retrovirology. 2015 Feb 10;12:15. doi: 10.1186/s12977-015-0135-y (PMC4335690; doi:10.1186/s12977-015-0135-y)
Supplement: Additional file 1: Table S1. — Demographic, HLA-typing, virological and clinical characteristics of patients. [file 12977_2015_135_MOESM1_ESM.pdf]

Sup. table S1. Demographic, HLA-typing, virological and clinical characteristics of patients

				HLA-I [a]		CD4	HIV infection		Treatment [e]	
ID	Gender	Age	Country	A*02	B*07	count [b]	Duration [c]	VL [d]	Duration	ART
						Cells/µl	*yr*	*Cp / mL*	*yr*	
1	M	37	US	+	-	895	<1	151	0	none
2	M	35	US	+	-	824	<1	125	0	none
3	M	30	US	-	-	468	9	6900	0	none
4	F	38	US	-	+	908	1	616	0	none
5	F	48	US	-	-	827	<1	47	0	none
6	F	40	US	-	-	805	<1	47	0	none
7	M	60	US	-	-	1176	1	14900	0	none
8	M	57	US	-	-	1145	<1	329	0	none
9	M	42	US	+	-	837	1	21	0	none
10	F	54	US	+	-	449	<1	16400	0	none
11	F	46	US	+	+	996	1	189	0	none
12	M	42	US	-	-	747	<1	1890	0	none
13	M	74	US	-	-	547	<1	2260	0	none
14	M	42	US	-	-	651	1	1127	0	none
15	M	70	US	+	-	731	1	8990	0	none
16	M	54	US	+	+	583	5	6940	0	none
17	M	39	US	+	-	653	2	90	0	none
18	M	50	US	+	-	617	3	184	0	none
19	M	63	US	-	-	647	3	248	0	none
20	F	54	US	-	-	922	<1	70	0	none
21	F	60	US	-	-	1036	<1	105	0	none
22	F	48	US	-	-	550	2	8400	0	none
23	F	47	US	-	-	818	2	6560	0	none
24	M	46	US	nd	nd	789	<1	7800	0	none
25	F	50	US	nd	nd	600	<1	1970	0	none
26	M	37	FR	+	nd	762	1	489	0	none
27	M	35	FR	+	nd	805	5	*nd*	0	none
28	M	30	FR	+	nd	478	7	<20	0	none
29	M	38	FR	+	nd	421	8	<20	6	AZT-3TC
30	F	48	FR	+	nd	432	21	<20	19	ETV-RAL-RTV-DRV
31	M	40	FR	+	nd	679	21	<20	19	RTV-ATV-FTC-TDF
32	M	60	FR	+	nd	431	29	<20	15	FTC-EFV-TDF
33	F	57	FR	+	nd	402	2	<20	2	ABC-3TC
34	M	42	FR	+	nd	467	6	<20	<1	FTC-TDF
35	M	54	FR	+	nd	610	27	<20	25	MVC-RAL-ABC-3TC
36	M	46	FR	+	nd	532	15	<20	15	RTV-ATV-FTC-TDF
37	M	42	FR	+	nd	564	9	<20	9	AZT-3TC-EFV
38	M	74	FR	+	nd	202	11	166	10	LPV/r-FTC-TDF
39	F	42	FR	+	nd	302	8	<20	8	RTV-ATV-FTC-TDF
40	M	70	FR	+	nd	377	7	<20	1	FTC-EFV-TDF
41	F	54	FR	+	nd	418	4	<20	3	FTC-EFV-TDF
42	F	39	FR	+	nd	18	<1	10165	<1	RTV-DRV-FTC-TDF
43	M	50	FR	+	nd	332	26	23	18	EFV-FTC-TDF
44	M	63	FR	+	nd	578	6	29	6	RAL-FTC-TDF
45	F	54	FR	+	nd	283	20	<20	14	RTV-DRV-FTC-TDF
46	M	60	FR	+	nd	606	14	<20	16	ABC-3TC-RTV-DRV

Sup. table S1. Demographic, HLA-typing, virological and clinical characteristics of patients

ID	Gender	Age	Country	HLA-I [a] A*02	HLA-I [a] B*07	CD4 count [b]	HIV infection Duration [c]	HIV infection VL [d]	Treatment [e] Duration	Treatment [e] ART
						Cells/µl	*yr*	*Cp / mL*	*yr*	
47	M	48	FR	+	nd	595	22	<20	20	RAL-RTV-DRV-ABC-3TC-TDF
48	M	47	FR	+	nd	270	26	26265	3	RTV-DRV-FTC-TDF
49	M	46	FR	+	nd	1854	23	<20	16	RTV-ATV-FTC-TDF
50	M	32	FR	+	nd	213	1	391	1	MVC-RTV-DRV-FTC-TDF
51	F	37	FR	+	nd	318	9	<20	2	LPV/r
52	F	40	FR	+	nd	613	9	27	3	ABC-3TC-RTV-ATV
53	F	38	FR	+	nd	380	2	49	<1	RTV-DRV-FTC-TDF
54	M	55	FR	+	nd	900	27	<20	16	RAL-NVP
55	M	76	FR	+	nd	523	23	<20	21	ETV-RAL-ABC-3TC
56	M	43	FR	+	nd	459	21	<20	16	RTV-DRV
57	M	47	FR	+	nd	509	13	<20	11	AZT-3TC-ABC
58	M	55	FR	+	nd	823	19	<20	19	ABC-3TC-RTV-ATV
59	M	57	FR	+	nd	1111	18	<20	17	RTV-DRV-ABC-3TC-ETV
60	M	52	FR	+	nd	669	24	60	14	RTV-DRV
61	F	57	FR	+	nd	1112	15	<20	8	RTV-ATV-FTC-TDF
62	F	49	FR	+	nd	441	14	<20	11	RTV-ATV
63	M	86	FR	+	nd	601	9	<20	8	RAL-ABC-3TC
64	M	48	FR	+	nd	429	26	<20	21	SQV-RTV-ATV
65	M	40	FR	+	nd	267	10	<20	7	RTV-DRV-ABC-3TC-MVC
66	F	29	FR	+	-	800	2	21	2	ABC-3TC-RTV-ATV
67	M	50	FR	+	-	341	23	<20	6	3TC-EFV-TDF
68	M	56	FR	+	-	508	24	975	0	none
69	M	48	FR	+	+	553	12	89	<1	ETV-FTC-TDF
70	M	23	FR	+	+	432	1	<20	1	RAL-FTC-TDF
71	M	59	FR	+	+	518	25	<20	5	RAL-FTC-TDF
72	F	48	FR	+	+	238	28	<20	22	MVC-RTV-DRV
73	M	45	FR	+	+	709	2	<20	1	RTV-DRV
74	F	34	FR	+	+	460	12	22	12	ABC-3TC-RTV-DRV
75	M	56	FR	nd	+	429	22	56	0	none
76	F	51	FR	nd	+	218	2	<20	2	RTV-DRV-FTC-TDF
77	M	42	FR	nd	+	236	8	<20	8	RAL-FTC-TDF
78	M	25	FR	nd	+	650	6	<20	2	FTC-EFV-TDF
79	M	43	FR	nd	+	597	25	<20	17	RAL-RTV-DRV-ATV-FTC-TDF
80	M	55	FR	nd	+	746	28	<20	0	none
81	M	43	FR	nd	+	653	14	<20	14	RTV-DRV-FTC-TDF
82	M	28	FR	nd	+	203	5	<20	2	RTV-DRV-FTC-TDF
83	M	57	FR	nd	+	854	15	101	15	MVC-FTC-TDF
84	M	50	FR	nd	+	423	26	<20	4	ETV-RAL-RTV-DRV-FTC-TDF
85	M	53	FR	nd	+	476	26	45	23	MVC-ETV-RAL
86	M	70	FR	nd	+	356	21	<20	15	ABC-3TC-EFV
87	M	22	FR	nd	+	25	<1	122633	0	none
88	M	51	FR	nd	+	746	27	<20	22	ATV-FTC-TDF
89	M	61	FR	-	nd	998	27	75	17	LPV/r-ABC-3TC
90	M	37	FR	-	nd	394	9	<20	4	FTC-EFV-TDF
91	F	41	FR	-	nd	619	15	35	15	RTV-DRV-FTC-TDF
92	M	63	FR	-	nd	345	26	57	19	RTV-DRV-FTC-TDF

Sup. table S1. Demographic, HLA-typing, virological and clinical characteristics of patients

ID	Gender	Age	Country	HLA-I [a] A*02	HLA-I [a] B*07	CD4 count [b]	HIV infection Duration [c]	HIV infection VL [d]	Treatment [e] Duration	Treatment [e] ART
						Cells/µl	*yr*	*Cp / mL*	*yr*	
93	F	39	FR	-	nd	440	14	<20	5	3TC-LPV/r
94	M	58	FR	-	nd	347	7	<20	7	AZT-3TC-LPV/r
95	M	32	FR	-	nd	462	3	84735	3	FTC-EFV-TDF
96	F	52	FR	nd	-	364	3	<20	3	ETV-FTC-TDF
97	M	50	FR	nd	-	360	20	<20	13	RAL-FTC-TDF
98	F	32	FR	+	-	820	4	<20	4	ABC-3TC-RTV-ATV
99	M	48	FR	+	-	400	9	<20	4	ETV-FTC-TDF
100	F	79	FR	+	-	187	<1	<20	<1	RAL-FTC-TDF
101	M	56	FR	+	-	571	29	<20	26	ETV-RTV-DRV
102	F	61	FR	+	-	611	22	<20	17	ABC-3TC-ATV
103	M	40	FR	+	-	363	2	<20	2	FTC-TMC 278-TDF
104	M	47	FR	-	+	654	20	<20	19	FTC-TDF
105	M	55	FR	-	+	646	19	<20	19	RAL-ABC-3TC-RTV-DRV
106	M	28	FR	-	+	901	2	<20	2	ABC-3TC-RTV-DRV
107	F	33	FR	-	+	129	<1	34521	<1	FTC-EFV-TDF
108	M	28	FR	-	+	1059	6	<20	5	ABC-3TC-ATV
109	M	48	FR	-	+	403	5	<20	5	RTV-ATV-FTC-TDF
110	M	33	FR	-	+	611	6	<20	5	RTV-ATV-FTC-TDF
111	M	40	FR	-	-	859	6	<20	3	DTG
112	M	62	FR	-	-	838	20	<20	20	ATV-FTC-TDF
113	F	43	FR	-	-	806	17	<20	16	MVC-ATV
114	M	42	FR	-	-	535	7	<20	7	EFV-FTC-TDF
115	M	39	FR	-	-	1231	10	<20	9	FTC-RTV-ATV
116	F	34	FR	-	-	1045	5	<20	1	FTC-TDF
117	F	29	FR	-	-	552	11	<20	11	ATV-FTC-TDF
118	F	59	FR	-	-	564	26	<20	16	RAL-ATV
119	M	65	FR	-	-	575	20	<20	18	FTC-EFV-TDF
120	M	49	FR	-	-	517	19	<20	19	ATV-FTC-TDF
121	M	43	FR	-	-	522	2	<20	2	FTC-TMC 278-TDF
122	F	65	FR	-	-	263	21	<20	19	ETV-RAL
123	F	51	FR	-	-	1104	28	<20	21	ABC-3TC-RTV-DRV
124	F	48	FR	-	-	644	25	<20	19	RAL-FTC-TDF
125	M	76	FR	-	-	537	30	<20	23	MVC-ETV
126	M	44	FR	-	-	579	<1	2951507	0	none

Individuals showing ASP-specific T cell responses (ASP responders) are highlighted in grey

[a] HLA-A*02 and –B*07 positive (+) or negative (-) status determined by genotyping for Pats.1 to 25. For Pats.26 to 126, HLA typing was determined by flow cytometry, and HLA type of ASP responders was confirmed by genotyping.

[b] Peripheral CD4+ T lymphocyte count at the time of study.

[c] Time since HIV-1 detection

[d] Copies of HIV-1 RNA per milliliter of plasma at the time of study.

[e] Treatment at the time of the study.

FTC, Emtricitabine; EFV, Efavirenz; ABC, Abacavir; NVP, Nevirapine; 3TC, Lamivudine; AZT, Zidovudine; RTV, Ritonavir; SQV, Saquinavir; TDF, Tenofovir disoproxil fumarate; ETV, Etravirine; ATV, Atazanavir; RAL, Raltegravir; MVC, Maraviroc; DRV, Darunavir; LPV/r, lopinavir/ritonavir; DTG, Dolutegravir; EVG, Elvitegravir; TMC 278, Rilpivirine

ID, identifier; ART, antiretroviral therapy; nd, not determined
