# Supplementary material for: The HIV-1 Antisense Protein (ASP) induces CD8 T cell responses during chronic infection
Source: Retrovirology. 2015 Feb 10;12:15. doi: 10.1186/s12977-015-0135-y (PMC4335690; doi:10.1186/s12977-015-0135-y)
Supplement: Additional file 2: Figure S1. — Antigens designed for detection of epitope-specific T cell responses provide global sequence coverage of HIV-1 ASP. (A) Overlapping peptides were designed using ASP sequences isolated from transmitted founder virus of an acute patient (WITO_TF1, red) and a HIV-1 lab strain (NL4-3, green). Peptides encoded by both strains are indicated in purple. Amino acid differences between peptides are highlighted in bold. Translated sequences are shown in the 5’ to 3’ direction, corresponding to the N- and C-termini of ASP, respectively. Sequences for ASP-YL9 and ASP-TL10 peptides are boxed. ni: not included, if the peptide could not be synthesized or did not pass NEP’s quality control standards. (B) Matrix design for subpools ASP peptides (A to J, and 1 to 9). Peptide number refers to the peptides listed in (A). Patients responding to peptide subpools are indicated along the x- and right y-axis. [file 12977_2015_135_MOESM2_ESM.pdf]

Suppl. Fig. S1

A.

```
                                Start    10        20        30        40        50
                                |        |         |         |         |         |
WITO_TF1         FKYLSIARTLAWSCLMPQTVSCNICCCASIALSRLLCCCTIPDNNSLACTVSVSDAAPIVLPAAPKNPR
NL4-3            .....T..I..............R.........K.F.........C.......IE..............

Peptide 1        FKYLSIARTLAWSCL
Peptide 2            SIARTLAWSCLMPQT
Peptide 3                TLAWSCLMPQTVSC
Peptide 4                   WSCLMPQTVSCNICC
Peptide 4b                  WSCLMPQTVSCNRCC
Peptide 5                       MPQTVSCNICCCASI
Peptide 5b                      MPQTVSCNRCCCASI
Peptide 6                            SCNICCCASIALSRL
Peptide 6b                           SCNRCCCASIALSKL
Peptide 7                                 CCASIALSRLLCCCTI
Peptide 7b                                CCASIALSKLFCCCTI
Peptide 8                                       LSRLLCCCTIPDNNSL
Peptide 8b                                      LSKLFCCCTIPDNNCL
Peptide 9                                            CCCTIPDNNSLACTV
Peptide 9b                                           CCCTIPDNNCLACTV
Peptide 10                                               IPDNNSLACTVSV
Peptide 11                                                 DNNSLACTVSVSDAA
Peptide 11b                                                DNNCLACTVSVIEAA
Peptide 12                                                     LACTVSVSDAAPIVL
Peptide 12b                                                    LACTVSVIEAAPIVL
Peptide 13                                                         VSVSDAAPIVLPAAP
Peptide 13b                                                        VSVIEAAPIVLPAAP
Peptide 14                                                             DAAPIVLPAAPKNPR
```

```
                       50        60        70        80        90        100       110
                       |         |         |         |         |         |         |
WITO_TF1         IVLPAAPKNPRNTAPSVTALFSLCTTLLFALVGAIPNGSIFTTLYLYNSLLQLSFIFPPPGLKVSFW
NL4-3            ............K..IP.................T...................L.S......I.DS

Peptide 15       IVLPAAPKNPRNTAP
Peptide 15b      IVLPAAPKNPRNKAP
Peptide 16           AAPKNPRNTAPSVTA
Peptide 16b          AAPKNPRNKAPIPTA
Peptide 17               NPRNTAPSVTALFSL
Peptide 17b              NPRNKAPIPTALFSL
Peptide 18 (ni)              TAPSVTALFSLCTTL
Peptide 18b                  KAPIPTALFSLCTTL
Peptide 19                               TTLLFALVGAIP
Peptide 19b                              TTLLFALVGATP
Peptide 20                                TTLLFALVGAIPNGSI
Peptide 21                                    ALVGAIPNGSIFTTL
Peptide 21b                                   ALVGATPNGSIFTTL
Peptide 22                                        AIPNGSIFTTLYLY
Peptide 22b                                       ATPNGSIFTTLYLY
Peptide 23                                           NGSIFTTLYLYNSLL
Peptide 24                                               FTTLYLYNSLLQLSF
Peptide 25                                                   YLYNSLLQLSFIFPP
Peptide 25b                                                  YLYNSLLQLSLISPP
Peptide 26                                                       SLLQLSFIFPPPGLK
Peptide 26b                                                      SLLQLSLISPPPGLK
Peptide 27                                                           LSFIFPPPGLKVSFW
Peptide 27b                                                          LSLISPPPGLKISDS
```

Suppl. Fig. S1 (continued)

```
                       110       120       130       140       150       160
                        |         |         |         |         |         |
WITO_TF1     FPPPGLKVSFWLLLLPPSLVNINPVIFDEQSICPLMGGAYIAFPTSCHMFITCFILHGSVILS--A
NL4-3        S......I.DS..........SS......HL.............F.....I......R..V.LPS

Peptide 28   FPPPGLKVSFWLLLL
Peptide 28b  SPPPGLKISDSLLLL
Peptide 29       GLKVSFWLLLLPPSL
Peptide 29b      GLKISDSLLLLPPSL
Peptide 30           SFWLLLLPPSLVNI
Peptide 30b           DSLLLLPPSLVNSSP
Peptide 31              LLLLPPSLVNINPVI
Peptide 32                  PPSLVNINPVIF
Peptide 33                   PSLVNINPVIFDEQSI
Peptide 33b                  PSLVNSSPVIFDEHLI
Peptide 34                        INPVIFDEQSICPLM
Peptide 34b                       SSPVIFDEHLICPLM
Peptide 35                            IFDEQSICPLMGGAY
Peptide 35b                           IFDEHLICPLMGGAY
Peptide 36                                QSICPLMGGAYIAFP
Peptide 37                                    PLMGGAYIAFPTSC
Peptide 37b (ni)                              PLMGGAYIAFPTFC
Peptide 38                                       GGAYIAFPTSCHMFI
Peptide 38b                                      GGAYIAFPTFCHMFI
Peptide 39                                           IAFPTSCHMFITCFI
Peptide 39b                                          IAFPTFCHMFIICFI
Peptide 40                                               TSCHMFITCFIL
Peptide 41                                                SCHMFITCFILHGSV
Peptide 41b                                               FCHMFIICFILHGRV
Peptide 42                                                    FITCFILHGSVILS--A
Peptide 42b                                                   FIICFILHGRVIVSL
```

```
                    160       170       180       stop
                     |         |         |         |
WITO_TF1     FILHGSVILS--AVLFQ--VLVPLLNQVLLNSCVELQ*KNSPPQLKVCITTSG
NL4-3        .....R..V.LPS...DPS..QV.............*........L.V.I..

Peptide 43   FILHGSVILS--AVLF
Peptide 44      HGSVILS--AVLFQ--VLV
Peptide 44b     HGRVIVSLPSVLFDP
Peptide 44c        VIVSLPSVLFDPSVL
Peptide 45          ILS--AVLFQ--VLVPLL
Peptide 45b             PSVLFDPSVLQVLL
Peptide 46               AVLFQ--VLVPLLNQVL
Peptide 47                   Q--VLVPLLNQVLLNSC
Peptide 47b                  DPSVLQVLLNQVLLNSC
Peptide 48                         PLLNQVLLNSCVEL
Peptide 48b (ni)                   VLLNQVLLNSCVEL
Peptide 49                            NQVLLNSCVELQ-K
Peptide 50                              VLLNSCVELQ-KNSPP
Peptide 51                                  SCVELQ-KNSPPQLKV
Peptide 52                                      LQ-KNSPPQLKVCITT
Peptide 53                                           SPPQLKVCITTSG
```

B.

	Sub.1	Sub.2	Sub.3	Sub.4	Sub.5	Sub.6	Sub.7	Sub.8	Sub.9	Responders
**Sub. A**	Peptide 1	Peptide 11	Peptide 22	Peptide 32	Peptide 42	Peptide 52	Peptide 13b	Peptide 27b	Peptide 42b	-
**Sub. B**	Peptide 2	Peptide 12	Peptide 23	Peptide 33	Peptide 43	Peptide 53	Peptide 15b	Peptide 28b	Peptide 44b	Pat.18, Pat.19
**Sub. C**	Peptide 3	Peptide 13	Peptide 24	Peptide 34	Peptide 44	Peptide 4b	Peptide 16b	Peptide 29b	Peptide 44c	-
**Sub. D**	Peptide 4	Peptide 14	Peptide 25	Peptide 35	Peptide 45	Peptide 5b	Peptide 17b	Peptide 30b	Peptide 45b	Pat.07
**Sub. E**	Peptide 5	Peptide 15	Peptide 26	Peptide 36	Peptide 46	Peptide 6b	Peptide 18b	Peptide 33b	Peptide 47b	Pat.19
**Sub. F**	Peptide 6	Peptide 16	Peptide 27	Peptide 37	Peptide 47	Peptide 7b	Peptide 19b	Peptide 34b		-
**Sub. G**	Peptide 7	Peptide 17	Peptide 28	Peptide 38	Peptide 48	Peptide 8b	Peptide 21b	Peptide 35b		-
**Sub. H**	Peptide 8	Peptide 19	Peptide 29	Peptide 39	Peptide 49	Peptide 9b	Peptide 22b	Peptide 38b		Pat.07
**Sub. I**	Peptide 9	Peptide 20	Peptide 30	Peptide 40	Peptide 50	Peptide 11b	Peptide 25b	Peptide 39b		Pat.09, Pat.21
**Sub. J**	Peptide 10	Peptide 21	Peptide 31	Peptide 41	Peptide 51	Peptide 12b	Peptide 26b	Peptide 41b		-
Responders	-	-	-	-	-	-	Pat.04	-	-	
