# Supplementary material for: The HIV-1 Antisense Protein (ASP) induces CD8 T cell responses during chronic infection
Source: Retrovirology. 2015 Feb 10;12:15. doi: 10.1186/s12977-015-0135-y (PMC4335690; doi:10.1186/s12977-015-0135-y)
Supplement: Additional file 3: Table S2. — Binding affinity and conservation of HLA-A*02- and HLA-B*07-restricted HIV-1 epitopes. [file 12977_2015_135_MOESM3_ESM.pdf]

Supplemental Table S2.Binding affinity and conservation of HLA-A*02- and HLA-B*07-restricted HIV-1 epitopes.

Restriction	Epitope	HIV-1 antigen	Peptide	aa	Location[1] genome	Location[1] antigen	HLA-I binding score[2] BIMAS	HLA-I binding score[2] SYFPEITHI	HLA-I binding score[2] NetMHC	Epitope conservation (%)[3] Group M	Epitope conservation (%)[3] Clade B
HLA-A*0201	Published[4]	Gag$_{p17}$	Gag-SL9	SLYNTVATL	1018	77	157	31	94	32	46
	Published[4]	Pol	Pol-IV9	ILKEPVHGV	3474	464	-	30	72	61	76
	Published[4]	Env	Env-KL9	KLTPLCVTL	6585	121	75	27	27	81	86
	Published[4]	Nef	Nef-LL9	LTFGWCFKL	9364	190	-	21	33	65	73
	Predicted	ASP	ASP-AL9	ALFSLCTTL	7759	62	-	-	20	42	87
	Predicted	ASP	ASP-SL10	SLCTTLLFAL	7750	65	285	-	25	35	80
	Predicted	ASP	**ASP-YL9**	**YLYNSLLQL**	**7686**	**88**	**723**	**28**	**7**	**85**	**90**
	Predicted	ASP	ASP-SL9	SLISPPPGL	7654	97	-	27	44	38	52
	Predicted	ASP	ASP-VV10	VLFDPSVLQV	7447	166	4125	-	12	<1	<1
	Predicted	ASP	ASP-VL9	VLLNSCVEL	7405	180	134	27	14	2.8	8
HLA-B*0702	Published[4]	Gag$_{p24}$	Gag-SV9	SPRTLNAWV	1231	148	-	19	19	92	94
	Published[4]	Gag$_{p24}$	Gag-TL9	TPQDLNTML	1327	180	80	21	-	68	94
	Published[4]	Gag$_{p2p7p1p6}$	Gag-YF10	YPLASLRSLF	2239	484	-	21	13	12	40
	Published[4]	Vpu	Vpu-QL10	QPIQIAIAAL	6065	2	80	22	82	<1	1
	Published[4]	Env	Env-AV10	APTKAKRRVV	7713	497	-	21	24	32	72
	Published[4]	Nef	Nef-RL9	RPMTYKAAL	9025	77	240	24	7	5	8
	Predicted	ASP	**ASP-TL10**	**TPNGSIFTTL**	**7711**	**78**	**80**	**24**	**76**	**15**	**35**
	Predicted	ASP	ASP-DL9	DPSVLQVLL	7438	169	80	24	-	<1	<1

[1] HXB2 nucleotide location and ASP or Gag aa position

[2] Relative scores from 3 algorithms predicting association of the peptide candidate with HLA-A*02 or HLA-B*07
Scores >80 for BIMAS, >24 for SYFPEITHI and <80 for NetMHC are predictive of a high HLA-I binding

[3] Determined using sequences from Los Alamos database; 4,418 (group M); 1,934 (clade B)

[4] References 40-42

- : not predicted; aa: amino acid
